# Nonalbumin Proteinuria in a Patient with Slowly Worsening Kidney Function

**DOI:** 10.34067/KID.0000000000000338

**Published:** 2024-03-26

**Authors:** Jennifer A. Schoonmaker, Margaret S. Ryan, Bhavna Bhasin-Chhabra

**Affiliations:** 1 Division of Nephrology and Hypertension, Mayo Clinic Arizona, Scottsdale, Arizona; 2 Division of Pathology, Mayo Clinic Arizona, Scottsdale, Arizona

**Keywords:** multiple myeloma, light chains, light chain tubulopathy, proteinuria, CKD

## Abstract

**Figure:**
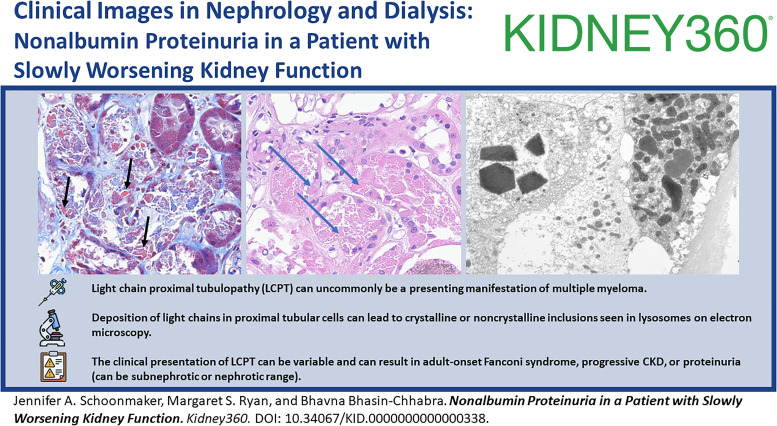

## Case Description

**Figure 1:**
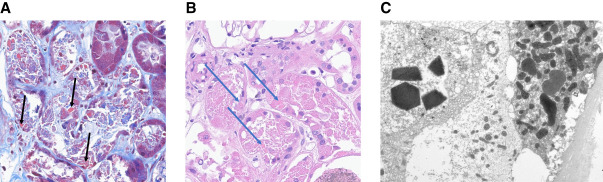
**Pathology images in LCPT.** (A) 40× trichrome stain and (B) 40× H&E: tubular epithelium with marked injury; intracellular crystalline inclusions are abundant within proximal tubular epithelial cells (arrows). (C) Electron microscopy: Within the tubular epithelium, pleomorphic and geometrically shaped lysosomes with crystalline substructure consistent with light chain accumulation are documented within the cytoplasm of many proximal tubule cells.

Sixty-eight-year old man with a history of hypertension, vitiligo, and hyperlipidemia presented to the clinic for concerns of renal dysfunction with slow uptrend in serum creatinine, which was noted to be 1.4 mg/dl on recent studies in contrast to 1.2 mg/dl 4 years ago. Urinalysis showed 1+ protein, small hemoglobin, negative red blood cells, and urine albumin to creatinine ratio of 73 mg/g. Urine protein to creatinine ratio was discordantly high at 1.35 g/g. Serum immunofixation studies showed monoclonal IgA kappa and high kappa:lambda ratio of 53.9. Kidney biopsy was undertaken which showed features of crystalline light chain proximal tubulopathy (LCPT) with marked tubular injury with proximal tubular intracellular crystalline inclusions on trichrome staining as demonstrated in Figure 1A (Trichrome staining), Figure 1B (Hematoxylin and Eosin staining), and electron microscopy (Figure 1C). Immunofluorescence showed monoclonal, kappa-restricted staining. Congo red staining was negative for amyloidosis. He was referred to hematology–oncology, and bone marrow biopsy was performed which showed plasma cell expansion of 30% consistent with multiple myeloma (MM).

## Discussion

Proteinuria can be a presenting sign of monoclonal gammopathy, and the discordance between urinalysis findings and urine protein to creatinine ratio is the hallmark finding of Bence Jones proteinuria as dipstick testing only detects albumin.^1^ With the presence of monoclonal immunoglobulins, bone marrow biopsy with at least 10% monoclonal plasma cells, and signs of end organ (renal) involvement as myeloma defining event, this patient met the criteria for a diagnosis of MM.^2^ Kidney involvement with MM can take the form of cast nephropathy, light chain, heavy chain or combined light and heavy chain deposition disease, immunotactoid or fibrillary glomerulopathy, proliferative glomerulonephritis with monoclonal immunoglobulin deposition, amyloidosis, or rarely LCPT.^3^

LCPT is characterized by deposition of light chains in the proximal tubules and is separated into two types: crystalline and noncrystalline morphology, with the former being more common. In normal circumstances, the kidney is able to reabsorb small amounts of the filtered free light chains using the megalin/cubilin receptor located on the proximal tubular apical surface. In LCPT, patients do not have normal metabolism of the light chains which then assume different shapes (polygonal, rhomboidal, rectangular, rod-shaped, or needlelike) and structures within lysosomes which can be visualized on electron microscopy. In the noncrystalline variant, the light chains deposit as droplets, granules, vacuoles, or fibrils in the proximal tubular cytoplasm. LCPT can also overlap with light chain podocytopathy and crystal storing histiocytosis in some cases. In addition to MM and monoclonal gammopathy of renal significance, LCPT has also been reported in association with chronic lymphocytic leukemia, lymphoma, and Waldenstrom macroglobulinemia.^4,5^

The clinical presentation of LCPT can be heterogenous with variable degree of proteinuria (subnephrotic or nephrotic range), adult onset of Fanconi syndrome (in a subset of patients), and different stages of CKD. Treatment is directed at the underlying clone, and renal remission depends heavily on hematological remission which may be achieved by stem cell transplantation after chemotherapy in select patients. Overall, patients who respond well to treatment are likely to have a favorable renal prognosis as well.^4,5^

## Teaching Points

- LCPT can uncommonly be a presenting manifestation of MM.
- Deposition of light chains in proximal tubular cells can lead to crystalline or noncrystalline inclusions seen in lysosomes on electron microscopy.
- The clinical presentation of LCPT can be variable and can result in adult-onset Fanconi syndrome, progressive CKD, or proteinuria (can be subnephrotic or nephrotic range).
